# Apolipoprotein C3 and apolipoprotein B colocalize in proximity to macrophages in atherosclerotic lesions in diabetes

**DOI:** 10.1194/jlr.ILR120001217

**Published:** 2020-12-13

**Authors:** Jenny E. Kanter, Karin E. Bornfeldt

**Affiliations:** 1 Department of Medicine, Division of Metabolism, Endocrinology and Nutrition, University of Washington Medicine Diabetes Institute, University of Washington School of Medicine, Seattle, WA, USA; 2 Department of Laboratory Medicine and Pathology, University of Washington Medicine Diabetes Institute, University of Washington School of Medicine, Seattle, WA, USA

**Figure 1:**
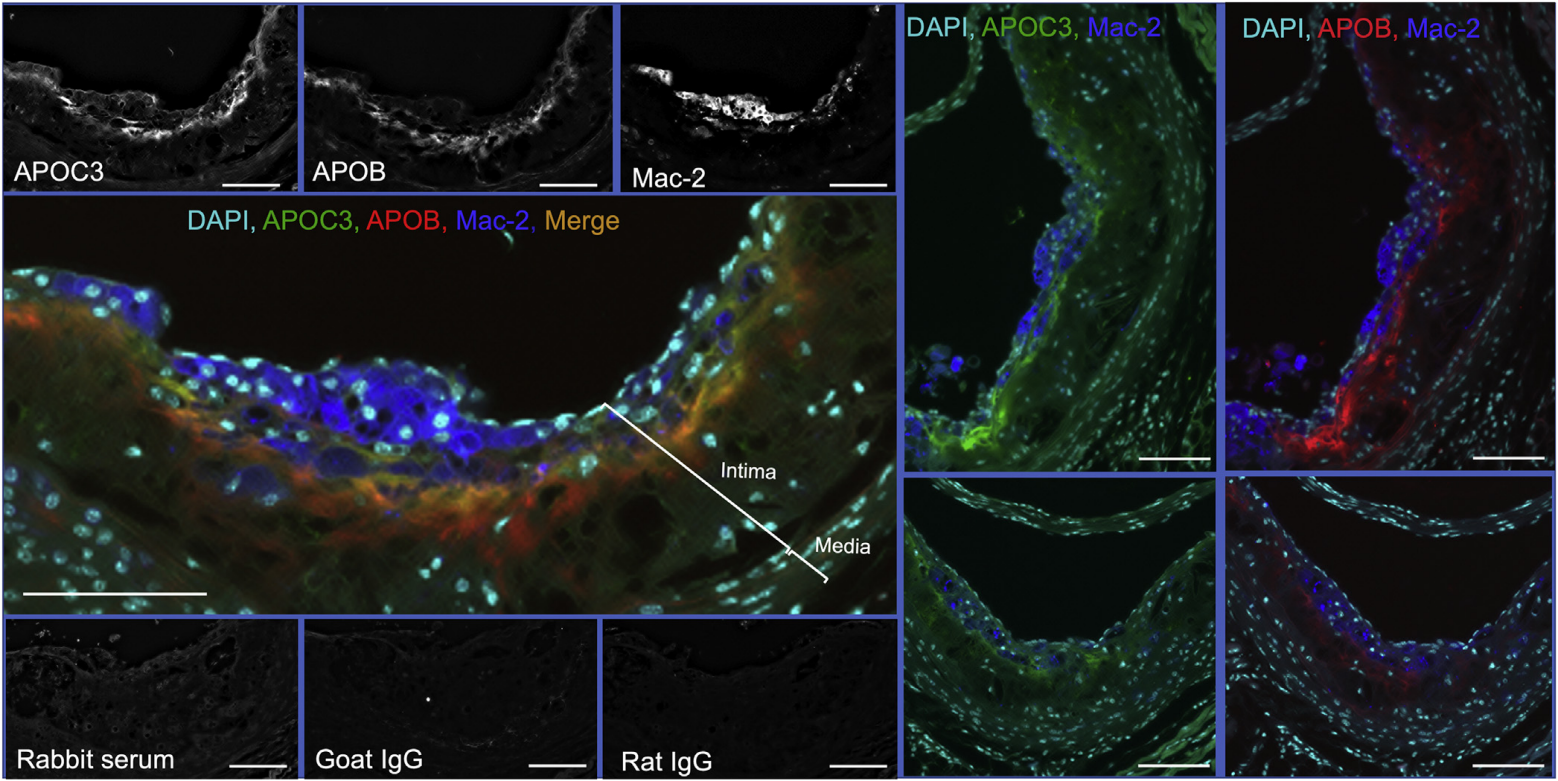

Increased serum levels of apolipoprotein C3 (APOC3) predict incident cardiovascular events and associate with coronary artery calcium in subjects with type 1 diabetes (T1D) (1, 2, 3). APOC3 plays a critical role in slowing clearance of triglyceride-rich lipoproteins and their remnant lipoprotein particles (4). Furthermore, mechanistic studies in a mouse model of T1D indicate a causal role for APOC3 in diabetes-accelerated atherosclerosis (1). Increased trapping of APOC3-containing lipoproteins in the artery wall is likely to contribute to the increased atherosclerosis associated with diabetes (1). These images of atherosclerotic lesions from the T1D mouse model of accelerated atherosclerosis (1) show that APOC3 immunoreactivity is present in the artery wall and substantially colocalizes with apolipoprotein B (APOB). Furthermore, APOC3 and APOB are localized in close proximity to lesional macrophages, as detected by positive Mac-2 immunoreactivity. APOC3 and APOB immunoreactivity was primarily found within the acellular extracellular matrix (indicated by a lack of nuclei) in the intima, deeper in the lesion than the macrophages. In this study, diabetes was induced using lymphocytic choriomeningitis virus in transgenic *Ldlr*^*−/−*^*; Gp*^*Tg*^ mice fed a high-fat diet for 12 weeks before induction of diabetes, followed by 4 weeks of diabetes (1). The hearts were fixed in 10% neutral-buffered formalin for 24 h, and the aortic sinus was sectioned at the site where all three leaflets were present. Aortic sinus cross sections were immunostained using a combination of an anti-APOC3 antibody (green), an anti-APOB antibody (red), an anti-Mac-2 antibody (blue), and DAPI (teal) to detect cellular nuclei (scale bars = 100 μm). Images from individual channels and individual negative controls in gray scale are shown to reveal the dynamic range of intensities. The atherosclerotic lesion (intima) and media are indicated by brackets. Lesions from two other diabetic mice are shown on the right (localization of macrophages with APOC3 and APOB is shown separately). The finding that APOC3 immunoreactivity colocalizes (indicated by orange/yellow areas) with APOB supports the hypothesis the APOC3-containing lipoprotein particles accumulating in lesions in the setting of diabetes may be remnant lipoprotein particles. The accumulation of these lipoprotein particles might exacerbate lesional macrophage accumulation and cardiovascular disease risk.

**EQUIPMENT:** Keyence All-in-One Fluorescence Microscope BZ-X800.

**REAGENTS:** Primary antibodies included rabbit-anti-APOC3 serum (kind gift from Ionis Pharmaceuticals, Inc; at 1:1000), biotinylated polyclonal goat anti-APOB (BAF3556, R&D Systems; at 1:50), and monoclonal rat anti-Mac-2 (CL8942AP, Cedarlane; at 0.5 μg/ml). Negative controls included rabbit serum (at 1:1000), biotinylated goat-IgG (BAF108, R&D Systems; at 1:50), and rat IgG (CLCR2A00, Cedarlane; at 0.5 μg/ml), followed by secondary antibodies. Each antibody was used alone to generate single-stained controls. Secondary antibodies included Alexa-647-streptavidin (modified to red digitally), Alexa-488 anti-rabbit (green), and Alexa-555 anti-rat (Invitrogen; modified digitally to blue). DAPI-containing mounting media was used to identify nuclei (Invitrogen; modified digitally to teal). Additional controls included single-stained sections for verification of spectral specificity in each channel (not shown).
